# Supplementary material for: Complexome profiling of the Chlamydomonas psb28 mutant reveals TEF5 as an early PSII assembly factor
Source: Plant Cell. 2025 Mar 17;37(6):koaf055. doi: 10.1093/plcell/koaf055 (PMC12164590; doi:10.1093/plcell/koaf055)
Supplement: koaf055_Supplementary_Data [file koaf055_supplementary_data.zip › psb28_interactive.zip!/plots/intensity/qualityPlots/peptides/PRPL9_Cre12.g556050.t1.2.html]

### Cre12.g556050.t1.2

Trivial name: PRPL9  
SeqCoverage [median]: 13.00  
Mass: 22.01 kDa  
  
 

| Sequence | MissedCleavage | Mass [Da] | wtIntensity | muIntensity | Score |
| --- | --- | --- | --- | --- | --- |
| IASEGILNQIR | 0 | 1212.68 | 0.00 | 912.15 | 76.22 |
|  |  |  | 0.0 | 912.2 |  |
